# Microscopic nuclei classification, segmentation, and detection with improved deep convolutional neural networks (DCNN)

**DOI:** 10.1186/s13000-022-01189-5

**Published:** 2022-04-19

**Authors:** Zahangir Alom, Vijayan K. Asari, Anil Parwani, Tarek M. Taha

**Affiliations:** 1 Department of Pathology, St. Jude Children’s Research Hospital, Memphis, TN USA; 2 Department of Electrical and Computer Engineering, University of Dayton, Dayton, OH USA; 3 Department of Pathology, The Ohio State University, Columbus, OH USA

**Keywords:** Digital pathology, Nuclei detection, Nuclei segmentation, DRCN, R2U-net, And UD-net

## Abstract

**Background:**

Nuclei classification, segmentation, and detection from pathological images are challenging tasks due to cellular heterogeneity in the Whole Slide Images (WSI).

**Methods:**

In this work, we propose advanced DCNN models for nuclei classification, segmentation, and detection tasks. The Densely Connected Neural Network (DCNN) and Densely Connected Recurrent Convolutional Network (DCRN) models are applied for the nuclei classification tasks. The Recurrent Residual U-Net (R2U-Net) and the R2UNet-based regression model named the University of Dayton Net (UD-Net) are applied for nuclei segmentation and detection tasks respectively. The experiments are conducted on publicly available datasets, including Routine Colon Cancer (RCC) classification and detection and the Nuclei Segmentation Challenge 2018 datasets for segmentation tasks. The experimental results were evaluated with a five-fold cross-validation method, and the average testing results are compared against the existing approaches in terms of precision, recall, Dice Coefficient (DC), Mean Squared Error (MSE), F1-score, and overall testing accuracy by calculating pixels and cell-level analysis.

**Results:**

The results demonstrate around 2.6% and 1.7% higher performance in terms of F1-score for nuclei classification and detection tasks when compared to the recently published DCNN based method. Also, for nuclei segmentation, the R2U-Net shows around 91.90% average testing accuracy in terms of DC, which is around 1.54% higher than the U-Net model.

**Conclusion:**

The proposed methods demonstrate robustness with better quantitative and qualitative results in three different tasks for analyzing the WSI.

## Introduction

Nowadays, computational pathology has become a trendy research area; therefore, this research field gains significant attention from both the research community and people working in clinical practice. Automatic nuclei classification, segmentation, and detection are very fundamental problems in Digital Pathology (DP), and prerequisites for various quantitative and qualitative analyses of different cancers, including routine colon cancer, breast cancer, drug development, and many more. The automatic nucleus classification, segmentation, and detection systems can significantly help unlock a cure faster for more diseases like cancer. Identification of the cell’s nuclei is the starting point to analyzing about 30 trillion cells, each of which contains a nucleus full of DNA within the human body. Accurate detection of cells can help the researchers to determine how to react to a cell for different treatments. As a result, the researchers can understand the underlying biological process of cell-level analysis in a clinical workflow. This solution can help ensure better planning for the treatment of patients, and it can accelerate disease identification and drug discovery processes. Therefore, computational pathology and microscopy images play an essential role in decision-making for disease diagnosis. These image analysis methods provide a wide range of information for computer-aided diagnosis (CAD) and enable a quantitative and qualitative analysis of images with a high throughput rate [1–3].

The proposed DL approaches can provide faster and more efficient image analysis results compared to the manual system currently used by the researchers and clinician-scientists. In addition, the system alleviates difficulty and requires repeated routine efforts [4]. The pathological images are very challenging to analyze manually; as a result, it can lead to large inter-observer variations [5]. On the other hand, CAD reduces the bias significantly and provides a characterization of diseases accurately [6]. Additionally, computational pathology gives a reproducible and rigorous measurement of pathological image features, which can be used for clinical follow-up. It may also help to study personalized medicine and treatment, which would significantly benefit patients. As a prerequisite of clinical practice of CAD, the nuclei classification, segmentation, and detection methods are considered for annotated image analysis with different DCNN based methods. These techniques provide various quantitative studies, including cellular morphology, size, shape, color, texture, and other imagenomics. However, these tasks are very challenging to achieve robust and accurate performance in pathological imaging for several reasons. First, the pathology and microscopy images contain background clutter with noise, artifacts (images are blur sometimes), low signal-to-noise ratio (SNR), and poor depth resolution. These degradations usually occur during image acquisition. Second, pathology images contain low contrast between the foreground and the background. Third, variations arise in terms of size, shape, and intercellular intensity of the nuclei or cell. Fourth, it can be observed very often that the nuclei of cells are partially overlapped with one another.

**Fig. 1:**
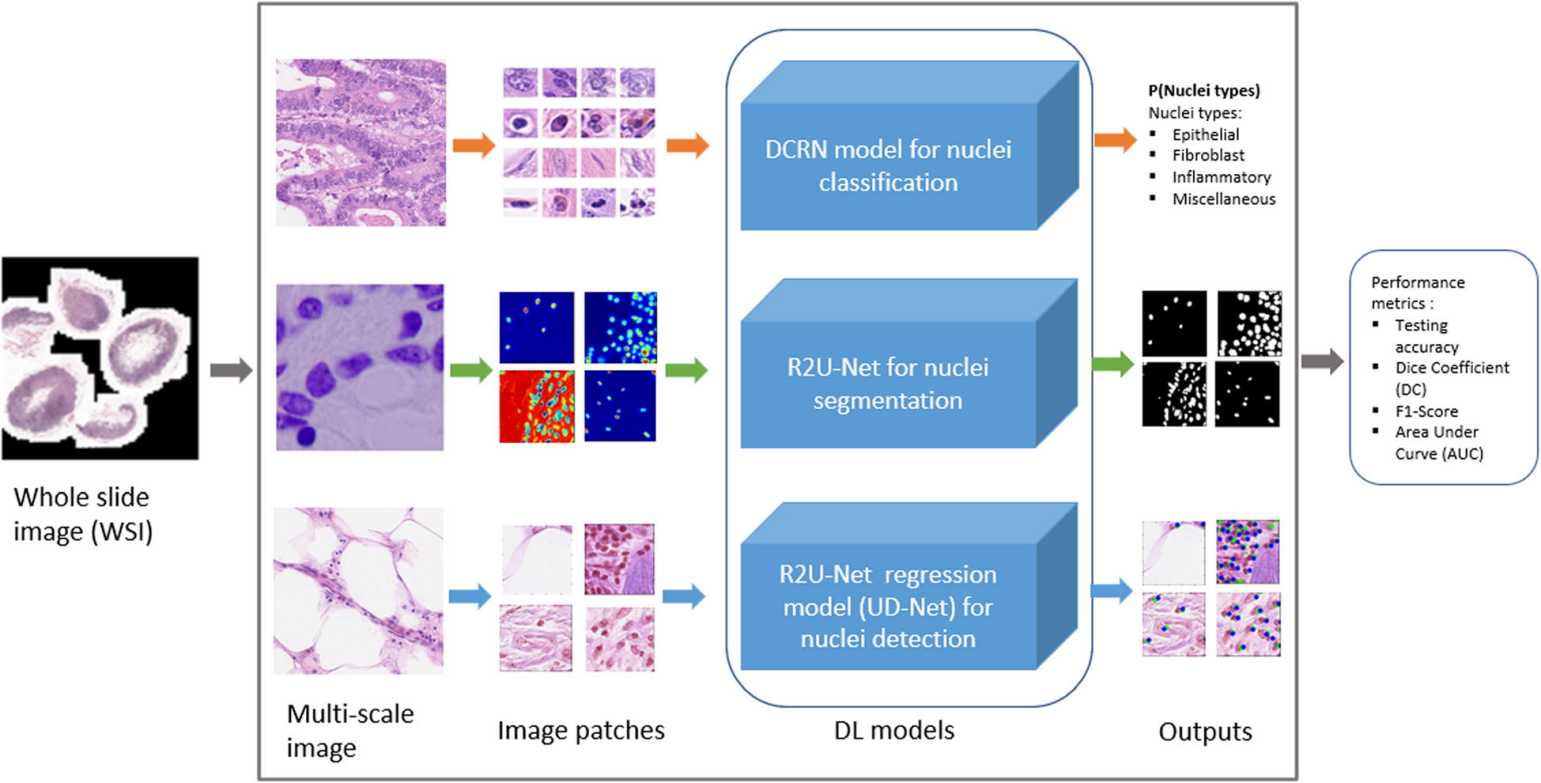
Schematic diagram of the proposed systems: the patches are extracted from the multi-scale slide as required. Three different DL models are applied for nuclei classification, segmentation, and detection tasks. Finally, the performance is evaluated with different performance metrics

Meanwhile, several methods have been proposed to tackle these issues with automatic nuclei classification, segmentation, and detection from pathological images. In the last few years, several surveys have been conducted, and CAD technologies in the field of biomedical imaging, including computational pathology, have been summarized [7–9]. These reviews briefly discuss different techniques related to image pre-processing, nuclei classification, segmentation, detection, and post-processing methods. One of the recently published papers discusses several techniques related to data acquisition and ground truth image preparation, image analysis, recognition, detection, segmentation, and survival analysis [10]. Another review was conducted on different approaches related to feature extraction, predictive modeling, and visualization in digital pathology applications [11]. A survey was conducted on nuclei detection, segmentation, and classification of hematoxylin and eosin (H&E) and immunohistochemistry (IHC) stained histopathology images. Due to the availability of annotated samples and computing power, the Convolutional Neural Network (CNN) successfully applied in different classification, segmentation, and detection problems, and shown state-of-the-art accuracy [12, 13]. For the classification task, the goal is to define the class probability from the input samples. For example, in binary-class breast cancer recognition problems, the system defines whether the input samples are either a benign or malignant class. Second, in most cases, deep CNN-based semantic segmentation techniques are used for nuclei segmentation, which describes the process of associating each pixel of an image with a class label and defining the proper contour of the target region from an input image. Third, in DCNN based cell detection task, the objectives are to identify the central or rectangular coordinate of specific cells and defining of the contour of a nucleus. However, due to the complex nature of pathological images, there are still several DL methods under development for even better accuracy. In this work, we applied three different improved DCNN models for nuclei classification, segmentation, and detection problems and considered each as an individual task. The overall project implementation schematic diagram is shown in Fig. 1. The contributions of this paper are summarized as follows:

- We proposed an improved model named the Densely Connected Recurrent Network (DCRN) and applied it to the nuclei classification task.
- An improved deep learning model called R2U-Net is applied to nuclei segmentation tasks.
- The R2UNet-based regression model named “UD-Net” is proposed and used for end-to-end nuclei detection tasks.

The experiments are conducted on three different publicly available datasets, and the results demonstrate superior performance compared to existing machine learning and recently published DL-based methods.

## Related works

In the last few years, several DCNN based approaches have been proposed and successfully applied to pathological image analysis problems and shown superior performance on different benchmark datasets for classification, segmentation, and detection [13]. In 2009, the image features, including shape, texture, and size of nuclei were considered to develop a classical method for nuclear pleomorphism grading for breast cancer detection tasks [14]. Malon et al. used a CNN for classifying mitotic, and non-mitotic cells using features that include the color, shape, and texture information [15]. The cancerous nuclei are classified from lymphocyte or stromal based on morphological features in H\&E stained for breast cancer image analysis problem, and a machine learning method was used to accurately segment tissue from the input samples [16]. A nuclei segmentation classification method was proposed using an AdaBoost classifier where the intensity, morphological, and texture features were used in [17]. However, recent studies have shown that the deep learning-based approaches demonstrate better classification accuracy for large-scale pathological image classification tasks [13]. In 2014, Wang et al. used hand-crafted features, and a cascaded ensemble CNN was applied for detecting nuclei and mitosis cells and achieved superior nuclei classification compared to classical machine learning methods [9]. Another deep learning-based approach was proposed for cell classification and was compared against a bag of features and canonical representations methods and achieved little better performance [18]. In 2017, a histopathological image classification approach was proposed using a support vector machine (SVM), AdaBoost, and DCNN methods. The experiment was conducted on four different H&E stained image datasets, namely the prostate, breast, renal clear cell, and renal papillary cancer cell detection tasks. The results demonstrate that the color-encoder deep network achieves the best performance out of nine individual classical methods and shows around 91.2% testing accuracy in terms of F1-score that is the highest testing accuracy to date [18]. For the very first time, we here introduce the Densely Connected Network (DCN) [19] and proposed a Densely Connected Recurrent Networks (DCRN) model for nuclei classification tasks.

For the nuclei segmentation task, a novel contour-based “minimum-model” cell detection and segmentation approaches were proposed in 2012 where a priori information was used to detect contours independent of their shape and achieved promising segmentation results [20]. The Nuclei membrane segmentation method was proposed using a CNN model from microscopic images in 2012 [21]. In 2015, Ronneberger et al. proposed a U-Net and applied this model for medical image segmentation tasks and achieved state-of-the-art performance [22]. A learning-based framework for robust and automatic nuclei segmentation was proposed that shows proper shape properties of nuclei in pathological images where a CNN base iterative region merging technique is applied. In 2016, a novel segmentation approach was exploited to separate individual nuclei by combining a robust selection-based shape sharing and a local repulsive deformable model, which were tested in several scenarios for pathological image segmentation and showed state-of-the-art performance against the existing machine and deep learning approaches [23]. A simple CNN model-based nuclei segmentation approach was proposed in 2017 named the CNN2 and CNN3 models for the different number of output classes. For the two-class model, the network was applied to classify pixels as inside or outside of the nuclei regions. On the other hand, for the three-class problem, the model was used for classifying pixels as belonging to the inside, outside, or boundary of nuclei regions [24]. In the same year, D. J. Ho et al. proposed a fully 3D-CNN method for nuclei segmentation method from 3D microscopy images [25]. A promising deep learning-based one-step contour aware nucleus segmentation approach was proposed with a fully convolutional neural network to segment the nuclei from corresponding boundaries simultaneously in 2018 [26]. A 3D Convolutional Network was used to perform cell nuclei detection and segmentation simultaneously in microscopic images, and the model was tested with two different datasets and achieved state-of-the-art accuracy in detection, and segmentation tasks [27]. However, for medical image segmentation problems, an improved version of the U-Net deep learning model was proposed in 2018, where recurrent residual modules were incorporated into the U-Net instead of forwarding convolutional layers. The model was evaluated on different modalities of medical imaging, including retinal blood vessel segmentation, skin cancer segmentation, and lung segmentation tasks, and achieved superior performance against U-Net, and SegNet [28]. To generalize the R2U-Net model, the R2U-Net model was applied for end-to-end nuclei segmentation tasks in 2018 [29]. In this study, a large-scale R2U-Net model is used for nuclei segmentation tasks on a larger dataset and achieved better performance.

For the nuclei detection task, two different approaches were primarily applied for nuclei detection: the first is detection-based counting, which requires a prior detection or segmentation in [30]. Another approach is a density estimation-based method that was used for nuclei detection without using segmentation methods in [31]. A framework with a supervised max-pooling CNN was trained to detect cell pixel regions using a Support Vector Machine (SVM) and outperformed against the hand-crafted feature-based approaches [32]. For nuclei detection, a stacked sparse autoencoder was used for non-nuclei and nuclei region detection with unsupervised fusion where a Softmax classifier was employed [33]. A CNN-based regression model was used for nuclei detection and counting, where a fully convolutional neural regression network model was used to identify the density map of nuclei from an input image with arbitrary size [34]. However, in this study, we propose a new R2UNet-based regression model for end-to-end nuclei detection from pathological images. The recurrent convolutional operations help the model learn and represent features better than the feed-forward convolutional operations and the robustness of the R2U-Net model has been discovered in several studies before [28].

## Proposed deep CNN models

### Densely connected recurrent convolutional network (DCRN)

**Fig. 2:**
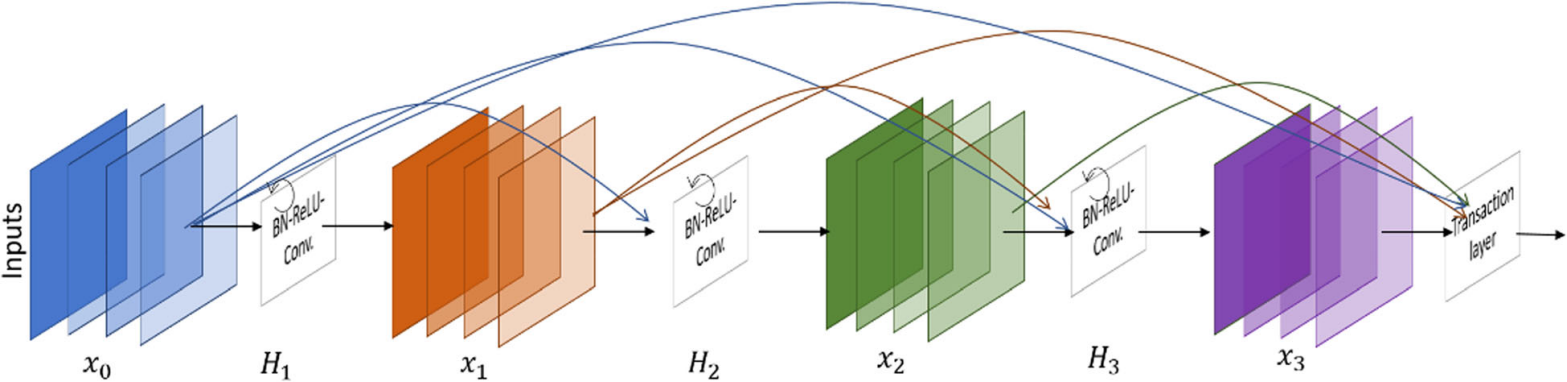
The Densely Connected Recurrent Network model with recurrent, convolutional, and transition blocks

According to the basic structure of Densely Connected Networks (DCN) in [19], the outputs from the previous layers are used as the input for the subsequent layers. This architecture ensures the reuse of the features inside the model and shows better performance for classification tasks [19]. In this implementation, we propose an improved version of the DCN, the Densely Connected Recurrent Network (DCRN) model, for nuclei classification. The DCRN is the building block of several densely connected recurrent blocks, and transition blocks are shown in Fig. 2.

According to the basic mathematical details of DenseNet explained in [19], the **l**^**th**^ layer receives all feature maps (**x**_**0**_, **x**_**1**_, **x**_**2**_⋯**x**_**l** − **1**_) from the previous layers as input:

1 $$ {\mathbf{x}}_{\mathbf{l}}={\mathbf{H}}_{\mathbf{l}}\left(\left[{\mathbf{x}}_{\mathbf{0}},{\mathbf{x}}_{\mathbf{1}},{\mathbf{x}}_{\mathbf{2}}\cdots {\mathbf{x}}_{\mathbf{l}-\mathbf{1}}\right]\right)\kern5.5em $$

where [**x**_**0**_, **x**_**1**_, **x**_**2**_⋯**x**_**l** − **1**_] is a concatenation of features from the **0**, ⋯⋯, **l** − **1** layers, and **H**_**l**_(∙) is a single tensor. Let’s consider the **H**_**l**_(∙) input sample from the **l**^**th**^ DCRN block containing **0**, ⋯⋯, **F** − **1** feature maps as inputs to the recurrent convolutional layers. The convolutional layer performs three consecutive operations including Batch Normalization (BN), followed by a ReLU and a **3** × **3** convolution. The (**i**, **j**) is a center pixel of a patch located in the input sample of the k^th^ feature named **H**_(**l**, **k**)_(∙). Additionally, the output of the network represents with **H**_**lk**_(**t**) for the l^th^ layer and the k^th^ feature map at time step *t*. The output can be expressed as in eq. (2).

2 $$ {\mathbf{H}}_{\mathbf{lk}}\left(\mathbf{t}\right)={\left({\mathbf{w}}_{\left(\mathbf{l},\mathbf{k}\right)}^{\mathbf{f}}\right)}^{\mathbf{T}}\ast {\mathbf{H}}_{\left(\mathbf{l}\right)}^{\mathbf{f}\left(\mathbf{i},\mathbf{j}\right)}\left(\mathbf{t}\right)+{\left({\mathbf{w}}_{\left(\mathbf{l},\mathbf{k}\right)}^{\mathbf{r}}\right)}^{\mathbf{T}}\ast {\mathbf{H}}_{\left(\mathbf{l}\right)}^{\mathbf{r}\left(\mathbf{i},\mathbf{j}\right)}\left(\mathbf{t}-\mathbf{1}\right)+{\mathbf{b}}_{\left(\mathbf{l},\mathbf{k}\right)} $$

**Fig. 3:**
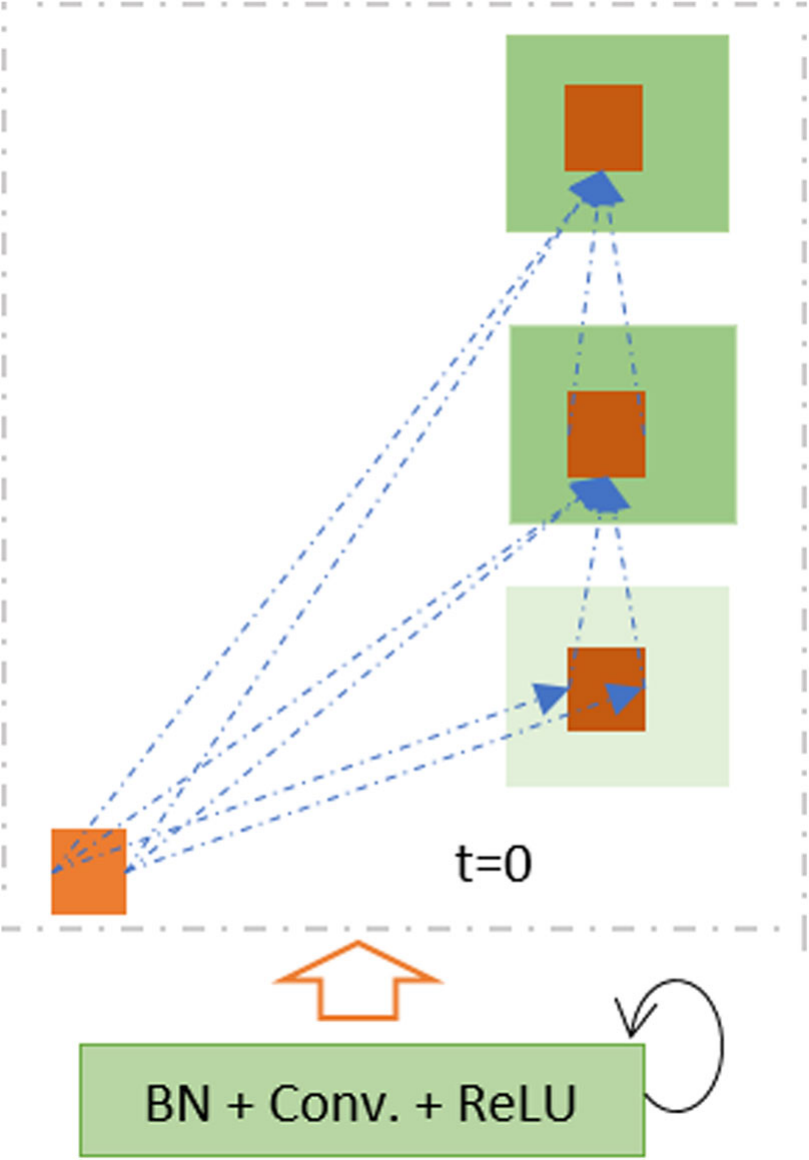
Unfolded recurrent convolutional layer for time step t = 2

Here, ${\mathbf{H}}_{\left(\mathbf{l}\right)}^{\mathbf{f}\left(\mathbf{i},\mathbf{j}\right)}\left(\mathbf{t}\right)$ and ${\mathbf{H}}_{\left(\mathbf{l}\right)}^{\mathbf{r}\left(\mathbf{i},\mathbf{j}\right)}\left(\mathbf{t}-\mathbf{1}\right)$ are the inputs to the standard convolution layers and the **l**^**th**^ recurrent convolution layers respectively. The ${\mathbf{w}}_{\left(\mathbf{l},\mathbf{k}\right)}^{\mathbf{f}}$ and ${\mathbf{w}}_{\left(\mathbf{l},\mathbf{k}\right)}^{\mathbf{r}}$ values are the weights of the standard convolutional layers and the recurrent convolutional layers of the l^th^ layer and k^th^ feature map respectively. The term **b**_(**l**, **k**)_ is the bias. The recurrent convolution operations are performed for the time steps *t* [35–37]. The pictorial representation of the recurrent convolution operations for **t** = **2** is shown in Fig. 3.

In the transition block, **1** × **1** convolutional operations are performed with BN followed by **2** × **2** average pooling layers. The DenseNet model consists of several dense blocks with feedforward convolutional layers and transition blocks, whereas the DCRN model uses the densely connected recurrent convolutional layers and transition blocks. The schematic diagram of recurrent convolutional layers is given in Fig. 3. For both DenseNet and the proposed DCRN models, we used four blocks with seven layers per block and a growth rate *k* of 12 in this study. The growth rate defines as a hyperparameter of DN. If the function *H*_*l*_ produces *k* output feature maps refer that *l*^*th*^ layer has *k* ∗ (*l* − 1) + *k*_0_ input features-maps where *k*_0_ is the number of channels in the input image. The model details for DenseNet and DCRN are shown in Table 1.

**Table 1:** The model architectures and the number of network parameters utilized for each model

Model	Tasks	t	Network architectures	# Parameters (mil.)
DenseNet	Classification	–	Blocks #4, layers#7, and growth rate # 12	1.228
DCRN	Classification	2	Blocks #4, layers#7, and growth rate # 12	1.228
R2U-Net	Segmentation	2	1➔ 32➔64➔128➔256➔128➔ 64➔32➔1	0.983
UD-Net	Detection	3	1➔32➔64➔128➔256➔128 ➔ 64➔32➔1	1.038

### R2U-Net

**Fig. 4:**
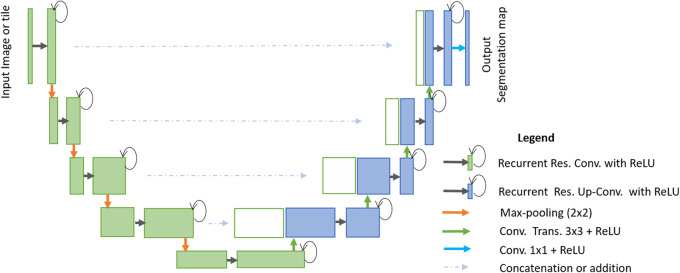
The end-to-end R2U-Net architecture where the green part refers to the encoding unit, and the blue part refers to the decoding units

**Fig. 5:**
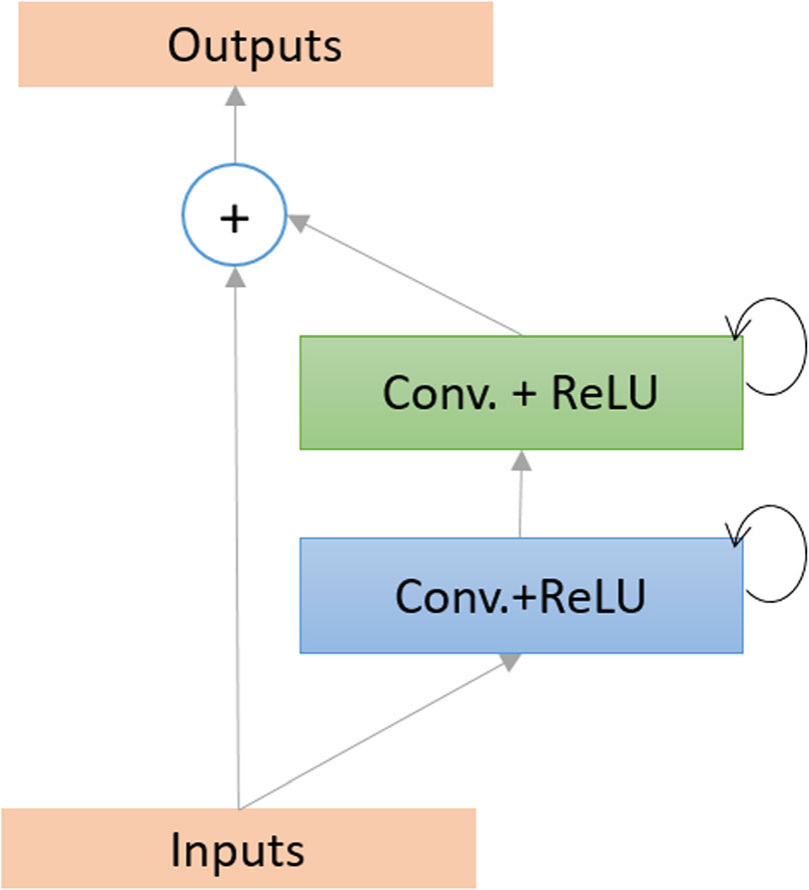
The recurrent residual unit (RRU) is used for DCRN, R2U-Net, and UD-Net models

We applied the R2U-Net model for nuclei segmentation for microscopic images in our previous study in 2018 [29]. However, we extended the nuclei segmentation tasks in this study by applying a large-scale R2U-Net model and achieved better performance. The R2U-Net model is an improved segmentation model developed based on U-Net [22], Recurrent Convolutional Neural Networks (RCNNs) [36], and the Residual Network (ResNet) [38]. The conceptual diagram of the R2U-Net model is provided in Fig. 4. The R2U-Net model consists of two main units that are encoding unit (shown in green) and the decoding unit (shown in blue). In both units, the recurrent residual convolutional operations are performed in the convolution blocks. A pictorial representation of the Recurrent Residual Convolutional Unit (RRCU) is shown in Fig. 5.

The recurrent operations are performed to different time steps, as shown in Fig. 3 for t = 2, which means one forward convolution layer followed by two recurrent layers are used in a convolutional unit. The feature maps from the encoding unit are concatenated with the feature maps from decoding units. The Softmax layer is used at the end of the model to calculate the pixel label class probability. The network architecture and model parameters for R2U-Net are given in Table 1.

### Regression model with R2U-Net

In general, for cell detection and counting problems, the ground truth masks are created with a single-pixel annotation method where the individually annotated single-pixel represents an entire cell. The dataset used in this study contains at least five to five hundred nuclei annotated manually with the center pixel annotation method. The annotations are then dilated with a 5 × 5 kernel, and a Gaussian distribution is generated for the dilated region. This regression model used the R2U-Net model to estimate the Gaussian density surface from the input samples instead of computing the classes directly or obtaining the pixel-level class probability. As the R2UNet-based regression model is used for nuclei detection, we named this model the University of Dayton Network (UD-Net). For any input sample, a density surface D(x) is generated based on a superposition of these Gaussians. The objective is to regress a density surface for the corresponding input image I(x). The target of the UD-Net model is minimized with the mean squared error between the predicted density and the target Gaussian density surface acts as the ultimate loss for the regression problem. In the testing phase, for a given input cell image I(x), the UD-Net model predicts the Gaussian density heat map D(x). In prior work, a CNN-VGG architecture-based regression model was proposed in 2015 [16, 39–41]. However, in this work, we propose a UD-Net regression model for nuclei detection tasks which is more powerful and robust compared to the existing methods.

### Model architectures

We used DenseNet and the DCRN models with similar architectures and a number of network parameters (around 1.22 M) for nuclei classification tasks, as shown in Table 1. The main difference between these two models is that feed-forward convolutional layers are used for DenseNet, whereas, for the DCRN model, we used a recurrent convolutional layer. For segmentation, we used the R2U-Net model with 0.98 M network parameters with t = 2. In addition, we used the UD-Net model with time step t = 3 that increases the number of network parameters to 1.038 M, which shows better testing performance. The architecture details of the R2U-Net and UD-Net regression and the number of network parameters are shown in Table 1.

## Experiments and results

To demonstrate the performance of the DCRN, R2U-Net, and R2U-Net-based regression (UD-Net) models, a five-fold cross-validation method has been considered for nuclei classification, segmentation, and detection tasks. The datasets for nuclei classification and detection tasks were taken from the recent study in [39], and the nuclei segmentation dataset was taken from the 2018 Data Science Bowl Grand Challenge dataset [42]. The average testing accuracies are reported in terms of Area Under ROC curve (AUC), Dice Coefficient (DC), and F1-score. For this implementation, the Keras [43], and TensorFlow [44] frameworks were used on a single GPU machine with 56G of RAM and an NVIDIA GEFORCE GTX-1080 Ti.

### Dataset for nuclei classification

**Fig. 6:**
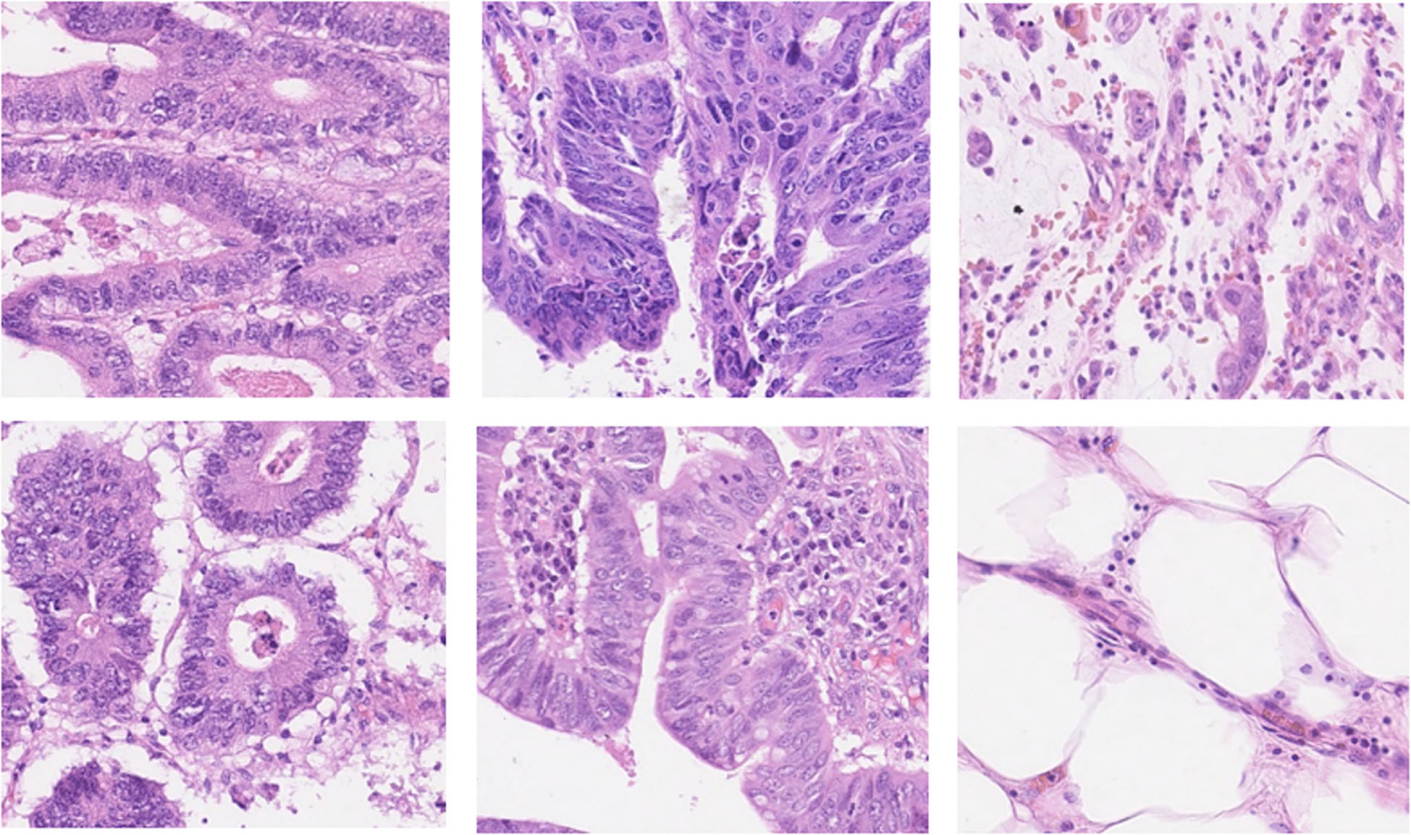
Randomly selected example images from the nuclei classification dataset

This dataset contains 200 annotated samples for classification and detection tasks, where 100 samples are used for classification, and the remaining 100 samples are used for detection. The actual sample size is 500 × 500 pixels. For both classification and detection tasks, randomly selected 80% of the samples are used for training, and the remaining 20% of samples are used for the testing phase. Some of the randomly selected samples for the nuclei classification task are shown in Fig. 6.

**Fig. 7:**
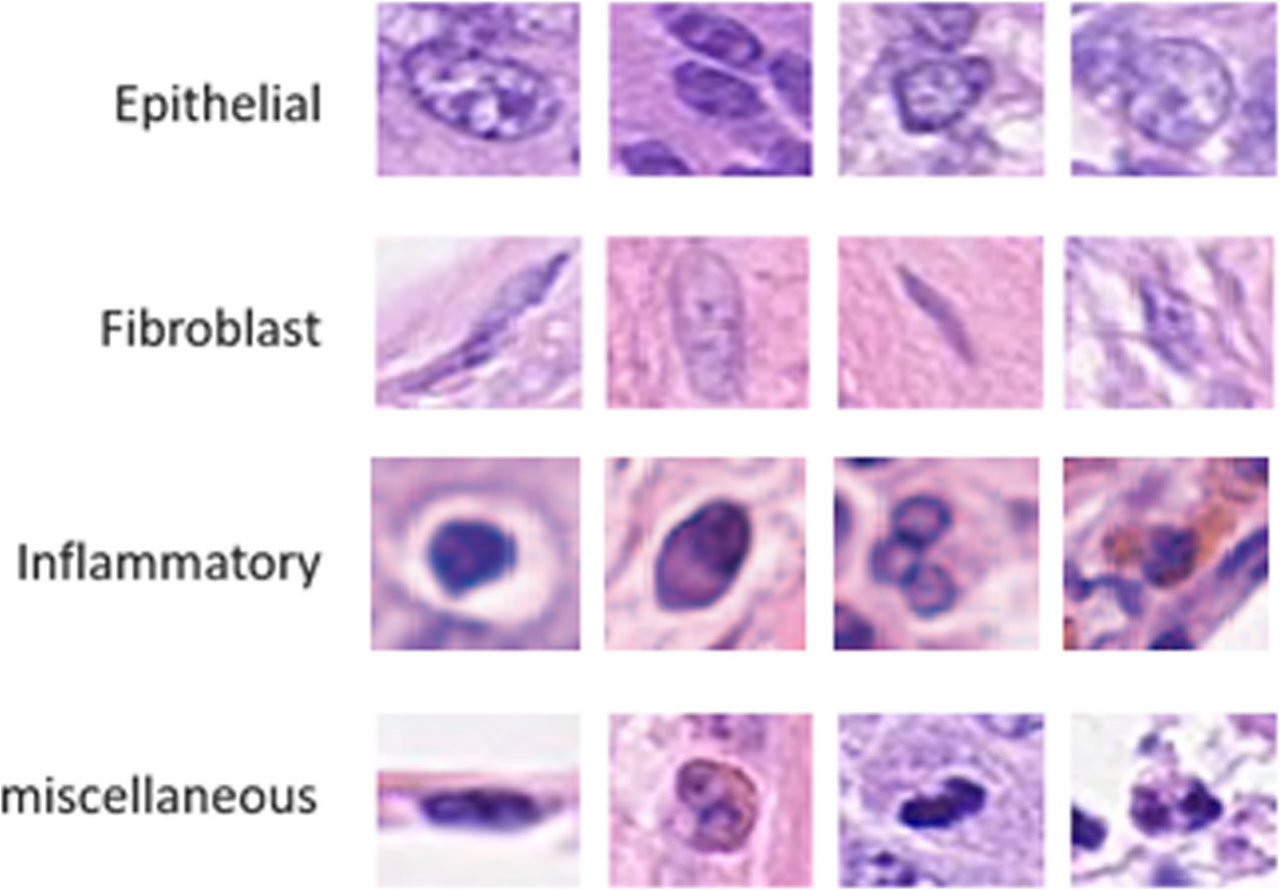
Randomly selected patches for four different types of nuclei of routine colon cancer

The dataset has four different classes of routine colon cancer for the classification task, including Epithelial, Fibroblast, Inflammatory, and miscellaneous. The samples are annotated with respect to the center pixel of the cell and provided as a MAT file. Each of the large patches (500 × 500 pixels) contains four different types of nuclei; however, we have observed that the large patches do not include all four types of nuclei cells in some cases. We have extracted patches with the size of 32 × 32 pixels to the center point of the cells from the large images. We have cropped 5295 patches for epithelial, 5424 patches for inflammatory, 4220 patches for fibroblast, and 1390 patches for miscellaneous. We have a total of 16,329 patches where 80% of samples are used for training, and the remaining 20% samples are used for validation as mentioned in [39]. The example patches are shown in Fig. 7.

### Dataset for nuclei segmentation

In 2018, the Data Science Bowl launched a competition to create a practical algorithm for automatic nucleus detection and segmentation. The nuclei segmentation database contains 735 images in total. The size of the samples is 256 × 256 pixels, where 650 images and their corresponding pixel-level annotation masks are released for training, and the remaining 65 samples for testing, respectively.

However, in this study, from the training set, 80% of the samples are used for training, and the remaining 20% are used for validation and testing. The number of training and testing samples is 536 and 134, respectively.

**Fig. 8:**
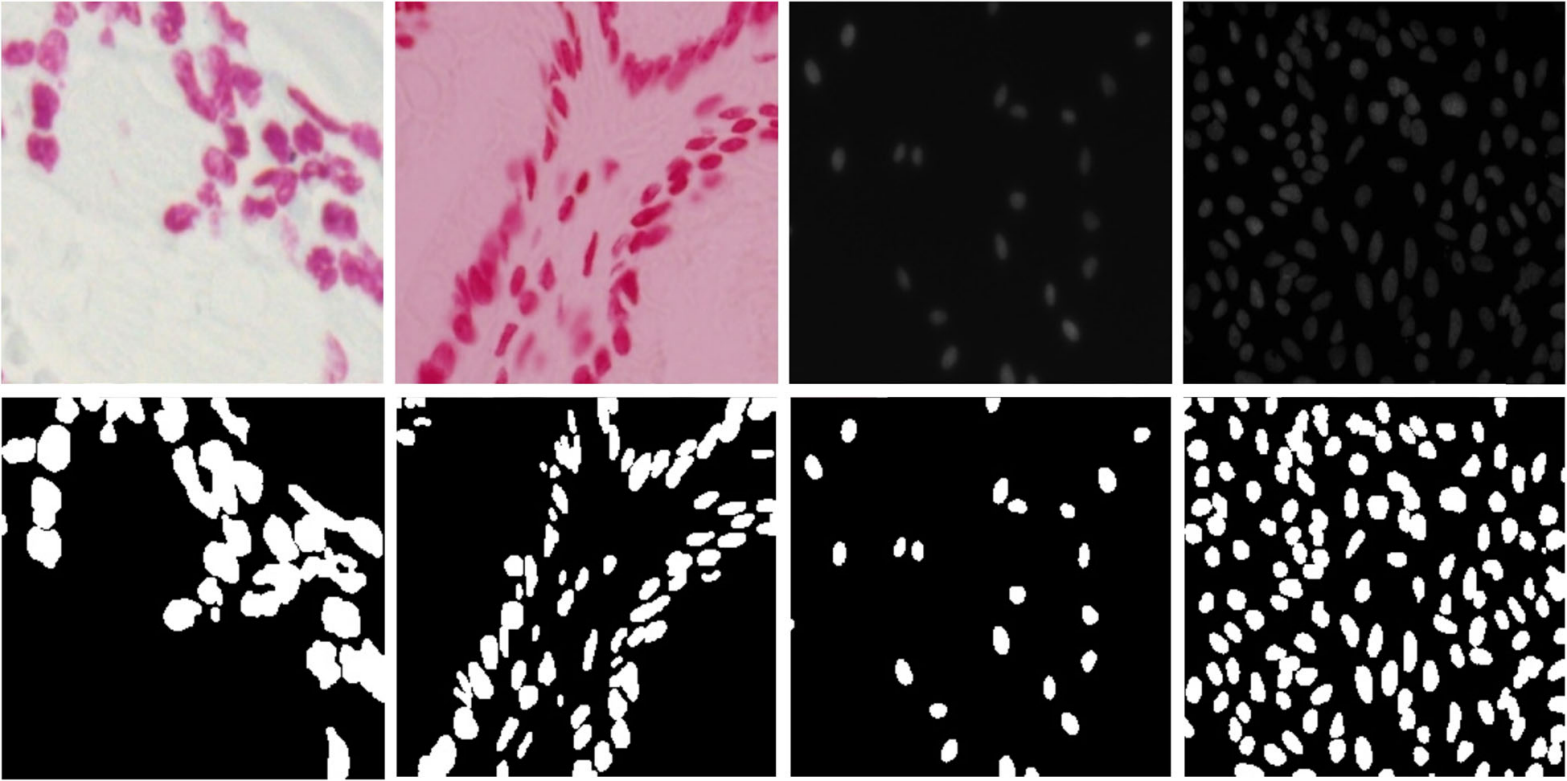
Example input images with segmentation masks: the first row shows input samples and the pixel label annotated masks are shown in the second row

This database contains both single and multichannel images; hence, we have converted all samples to gray-scale representation. Figure 8 shows the input samples in the first rows and corresponding ground truth masks in the second row.

### Database for nuclei detection

**Fig. 9:**
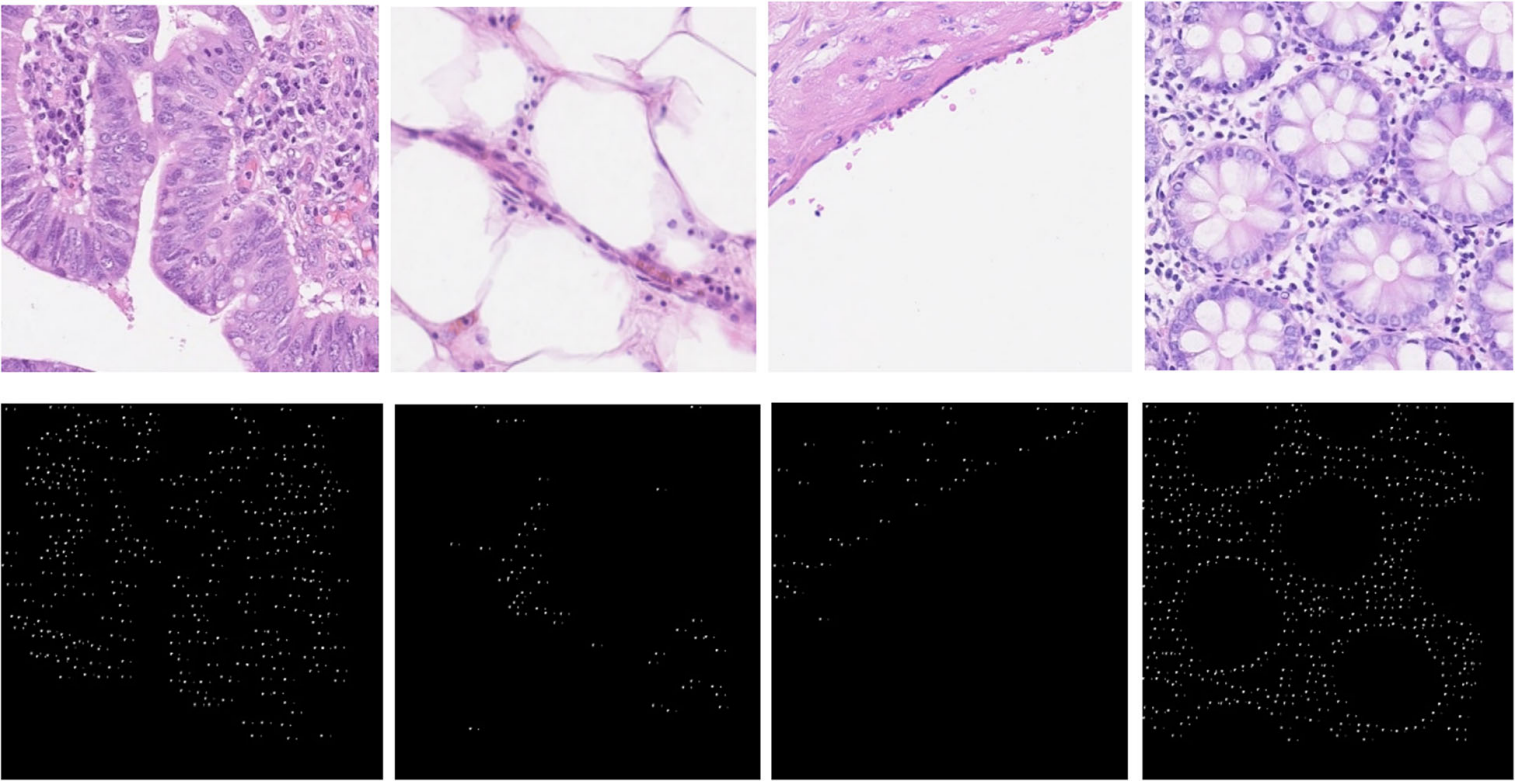
Randomly selected input imagesare shown in the first row and corresponding dilated masks with 3 × 3 kernels are shown on the second row for nuclei detection tasks

The nuclei detection database contains 100 samples and 100 masks with single-pixel annotation [33, 39, 45]. The original size of the database samples is 500 × 500. Some of the randomly selected samples and corresponding dilated masks are shown in Fig. 9. For nuclei detection, we extracted the non-overlapping patches with a size of 96 × 96 pixels from the input samples and corresponding masks. We used a total of 4392 non-overlapping patches and maks. Of these patches, around 80% are used for training, and the remaining 20% are used for testing.

### Evaluation metrics

The performance of the models for nuclei cell detection tasks is evaluated with different performance metrics, including precision, recall, and F1-score, which are stated in eqs. (3) through (5). The True Positive (TP) refers to the number of nuclei cells correctly detected with respect to the ground truth. In contrast, False Positive (FP) represents the number of detected nuclei not in the ground truth. The number of ground truth nuclei cells that are un-detected are called False Negatives (FNs). The mathematical representation of precision, recall, and F1-score are shown in the following expression as follows:

3 $$ \mathrm{precision}=\mathrm{TP}/\left(\mathrm{TP}+\mathrm{FP}\right) $$

4 $$ \mathrm{recall}=\mathrm{TP}/\left(\mathrm{TP}+\mathrm{FN}\right) $$

5 $$ \mathrm{F}1-\mathrm{score}={2}^{\ast}\left({\mathrm{recall}}^{\ast}\mathrm{precision}\right)/\left(\mathrm{recall}+\mathrm{precision}\right) $$

### Training methods

For nuclei classification tasks, the DenseNet and DCRN are used with similar architecture and a number of network parameters with a five-fold validation approach. And for training both models, we used a stochastic gradient descent (SGD) optimization method with a learning rate of 0.001, a weight decay of 1 × 10–4, a momentum of 0.9, and cross-entropy loss. The models are trained for 100 epochs with batch size 32. For the segmentation task, we applied the Dice Coefficient (DC) and Means Squared Error (MSE) loss. The DC is expressed in eq. (6), where GT refers to the ground truth, and SR refers to the segmentation result.

6 $$ \mathrm{DC}=2\ \frac{\left|\mathrm{GT}\cap \mathrm{SR}\right|}{\left|\mathrm{GT}\right|+\left|\mathrm{SR}\right|} $$

Another metric is used to evaluate the performance of the segmentation algorithm is the MSE as defined in eq. (7). In this case, Y represents ground truth and $\hat{\mathrm{Y}}$ represents the predicted outputs for an input sample with height *h* and width *w* where *n = h × w*.

7 $$ \mathrm{MSE}=\frac{1}{\mathrm{n}}\ \sum \limits_{\mathrm{i}=1}^{\mathrm{n}}{\left({\mathrm{Y}}_{\mathrm{i}}-{\hat{\mathrm{Y}}}_{\mathrm{i}}\right)}^2 $$

We trained a segmentation model with 250 epochs and used an Adam optimizer with a learning rate of 2 × 10^− 4^ and a batch size of 16. Finally, for detection with the UD-Net regression model, we used the Adam optimizer with a learning rate of 2 × 10^− 4^ and measured mean squared error (MSE). The UD-Net model is trained for 500 epochs and with a batch size of 64.

## Results and discussion

### Nuclei classification

**Fig. 10:**
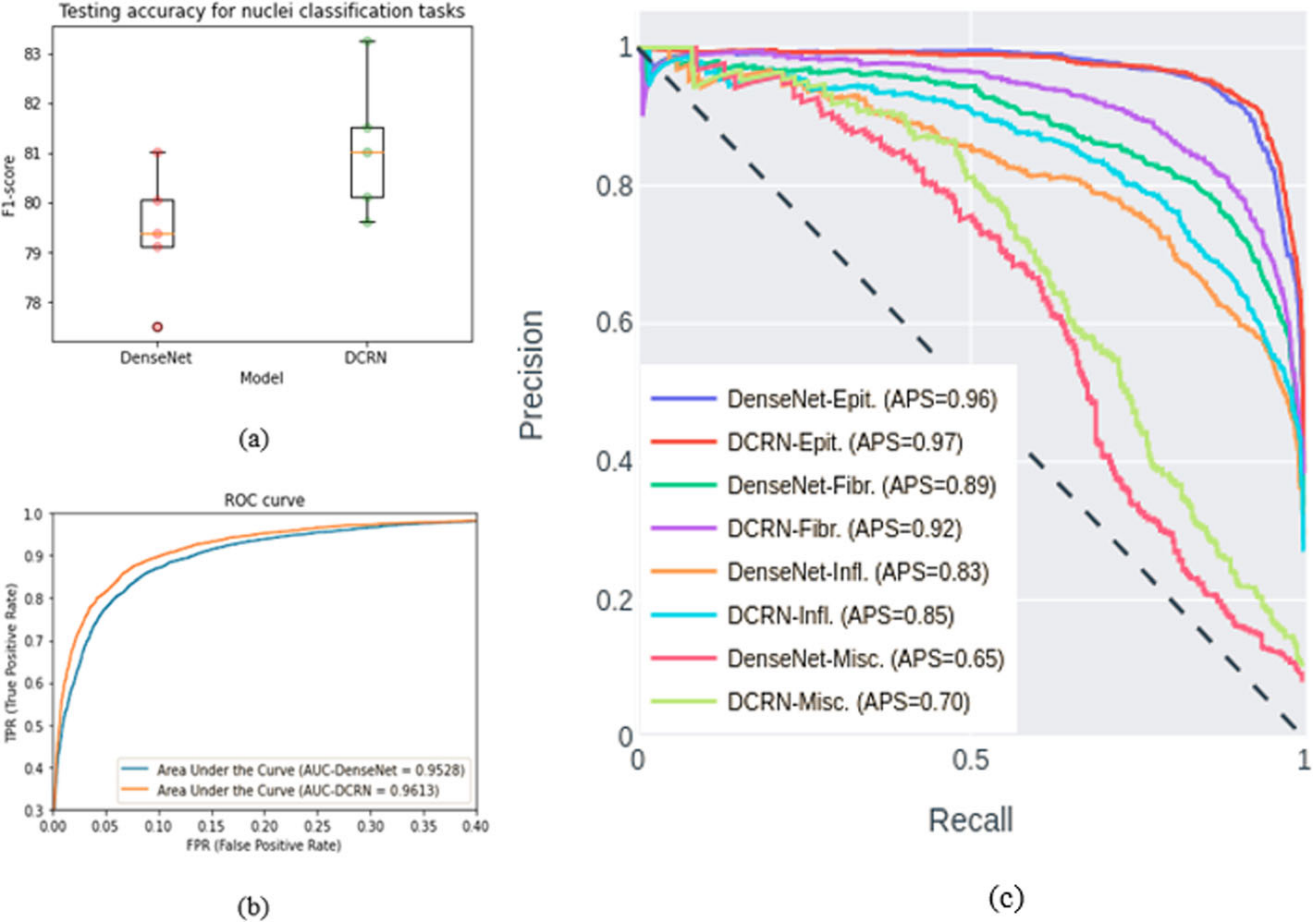
Results for nuclei classification model. **a** Box plot for testing F1-score. **b** Area under the ROC curve with average AUC and (b) precision-recall curve with Average Precision Score (AVS) of the DenseNet and DCRN models for nuclei classification tasks

We tested both DenseNet and DCRN models with the same setup with a five-fold validation method and achieved an average of 79.41 ± 1.16 percentage and 81.11 ± 1.27 percentage testing accuracy in terms of F1-score, respectively. The box-plot of the testing F1-score of the DenseNet and the DCRN models are shown in Fig. 10 (a), respectively. The DCRN outperformed the DenseNet model in most of the trials. The comparison against the existing nuclei classification methods is shown in Table 2; the proposed DCRN shows around 1.7% superior performance when compared against the DenseNet and observed a significant improvement over other existing methods. In addition, Fig. 10(b) demonstrates the ROC curve with Area Under the Curve (AUC) for both models. First, the False Positive Rate (FPR) and True Positive Rate (TPR), and AUC are calculated from the predicted outputs for four classes from both models. Then, the ROC curve is generated from these metrics where the DCRN shows 0.86% better AUC than the DenseNet. In addition, the precision versus recall curves with Average Precision Score (APS) is shown in Fig. 10(c), for demonstrating the performance of the individual class. For all four categories, the proposed DCRN shows better performance compared to the DenseNet model.

**Table 2:** Nuclei classification accuracy and comparison against other machine learning and deep learning methods

Methods	Average F1-score	AUC
CRImage [16]	0.488	0.684
Super-pixel descriptor [40]	0.687	0.853
SoftMax CNN + SSPP [39]	0.748	0.893
SoftMax CNN + NEP [39]	0.784	0.917
DenseNet [19]	0.794	0.9523
Proposed (DRCN)	0.811	0.9612

**Fig. 11:**
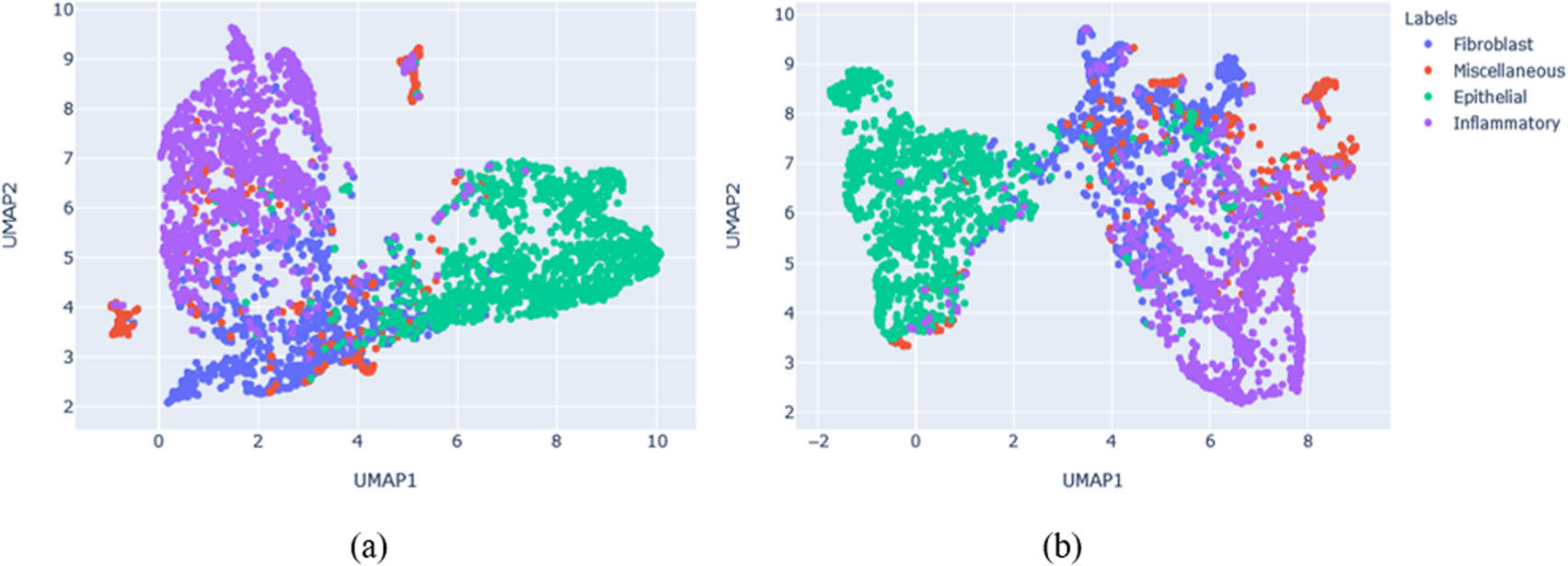
Unsupervised analysis. **a** Clustering of deep features extracted with DenseNet. **b** Clustering of deep features extracted with DRCN

Furthermore, the deep features have been extracted from the bottleneck layer from both models for 3967 testing samples. The dimension of feature representation is (3967 × 4 × 384). Then, a global average pooling is performed to generate the vector representation of (3967 × 384). Finally, the uniform Manifold Approximation and Projection (UMAP) is applied for dimensionality reduction, and clustering the features [46]. The clustering results for features extracted with the DenseNet and DCRN are shown in Fig. 11(a) and (b) respectively. From the plots, it can be clearly observed that the UMAP shows better clustering for four different types of nuclei with DCRN features when compared to the DenseNet. These results clearly demonstrate the robustness of our proposed DCRN model over the DenseNet for nuclei classification tasks.

### Nuclei segmentation

**Fig. 12:**
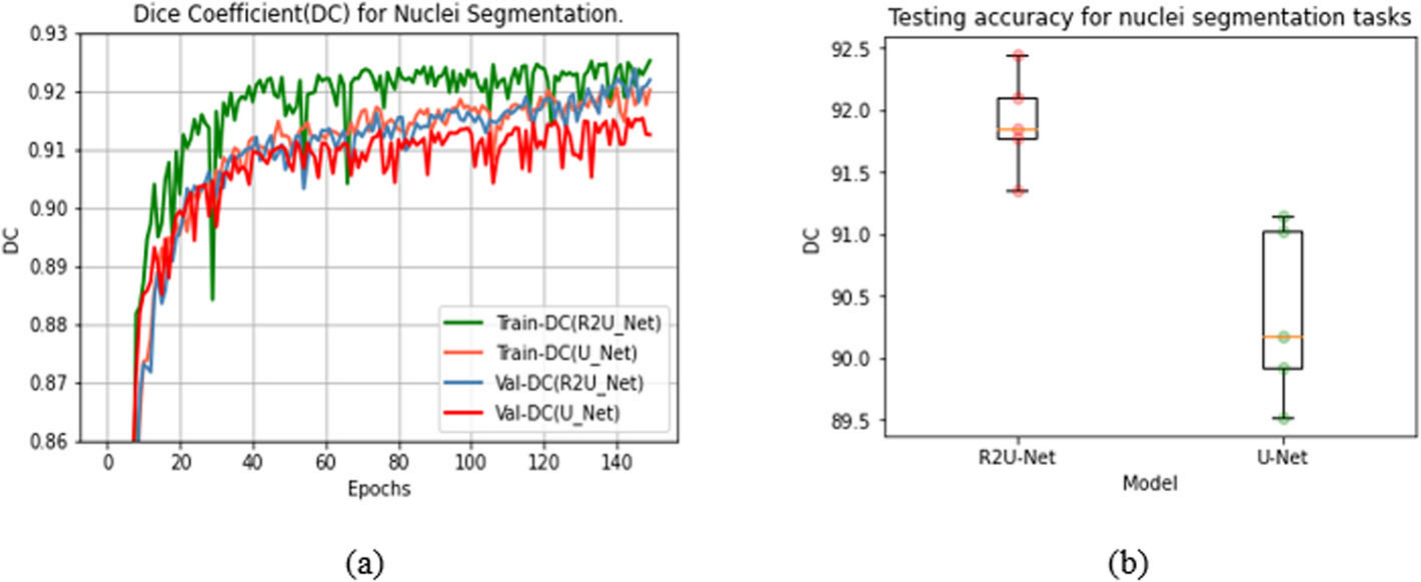
Results for nuclei segmentation model. **a** Training and validation DC for the best R2U-Net and U-Net models. **b** Box plot of testing DC score for five-fold validation

We used a simple R2U-Net model with only 0.983 million network parameters and considered the DC for monitoring the training progress and measuring the performance during the testing phases. From the experiments, we observed that the model converged after 100 epochs; however, the training and evaluation continued until 150 epochs to ensure better convergence, considering the lack of the number of samples available for training. In the testing phase, we achieved an average of (90.36 ± 0.633)% and (91.90 ± 0.364)% testing accuracy in terms of DC score with U-Net and R2U-Net models respectively. Figure 12(a) shows the training and validation DC for both U-Net and R2U-Net models for 150 epochs. The results demonstrate that the R2U-Net model learned better compared to the U-Net model during the training process. Figure 12(b) shows the box plot of testing DC score for five-fold validation. The R2U-Net shows a 1.54% better average DC score compared to the U-Net model for nuclei segmentation tasks.

#### Qualitative analysis

**Fig. 13:**
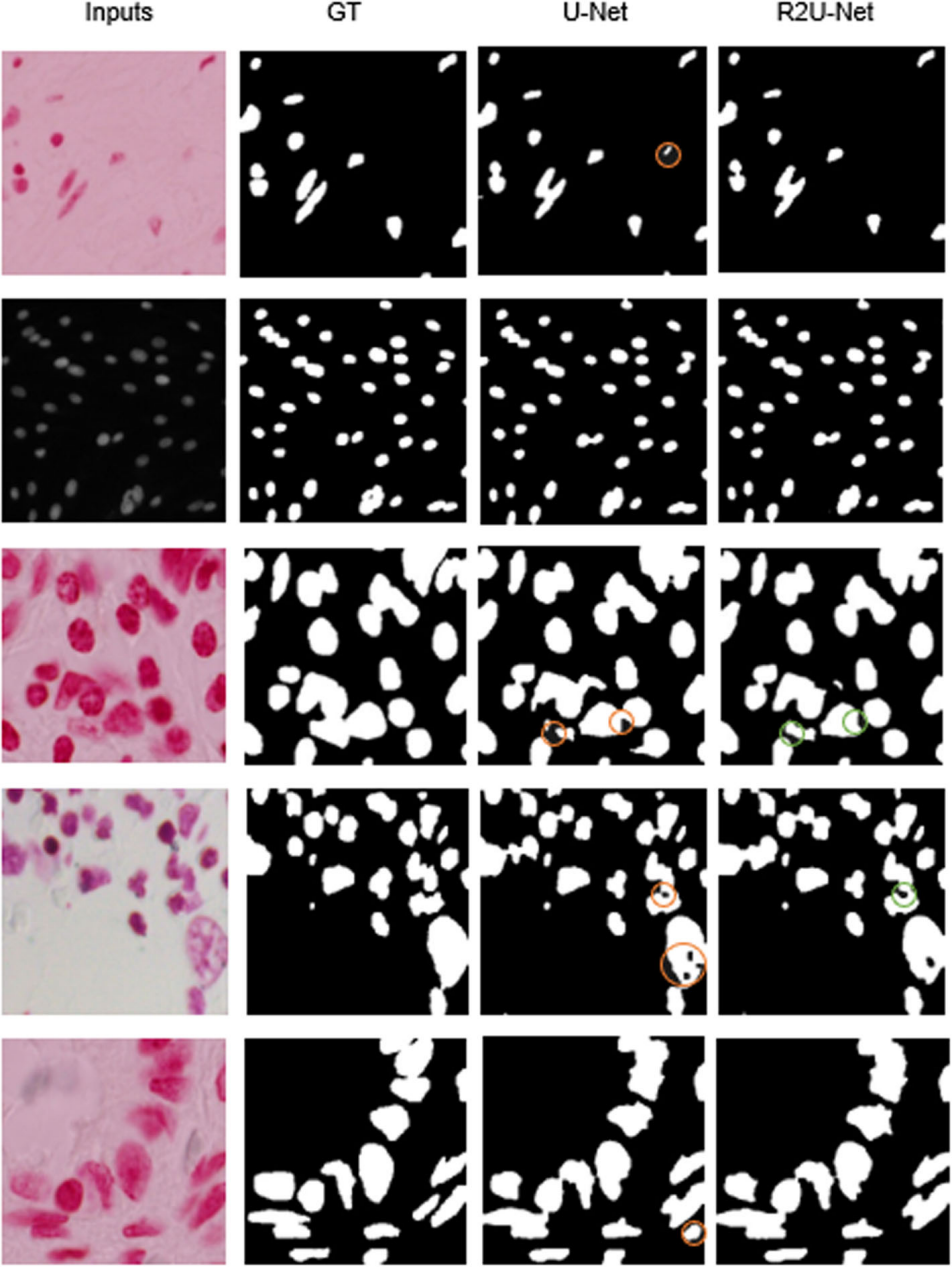
Qualitative results for both U-Net and R2U-Net models for nuclei segmentation, the first column shows the input samples, the second column shows the corresponding ground truth (GT) masks, the third column shows the outputs from U-Net and the fourth column shows the outputs of R2U-Net model. The orange circles show the false detection by the U-Net model

Figure 13 shows some example outputs when using the U-Net and R2U-Net models for nuclei segmentation tasks where the first column shows the input images, the second column shows the ground truth masks for the corresponding input samples, the third column shows the outputs of the U-Net model, and the fourth column represents the outputs for R2U-Net model. The proposed R2U-Net segmentation model shows better quantitative results compared to the U-Net model during the testing phase. We also observed that the input samples in the first row in the third column show the false detection, which is indicated with an orange circle. In contrast, the R2U-Net shows very accurate segmentation results like ground truth in the second column. Likewise, we can observe the same false detection results in the last rows. In the fourth row, the black regions appear in the nuclei regions, which are false negative. However, the R2U-Net model shows accurate segmentation output in this case. The U-Net model fails to show the isolated nucleus marked with an orange circle, whereas the R2U-Net successfully segmented and separated the individual nucleus in the third row. Thus, the segmentation results demonstrate the robustness of the R2U-Net model for nuclei segmentation tasks compared to the U-Net model.

### Nuclei detection

**Fig. 14:**
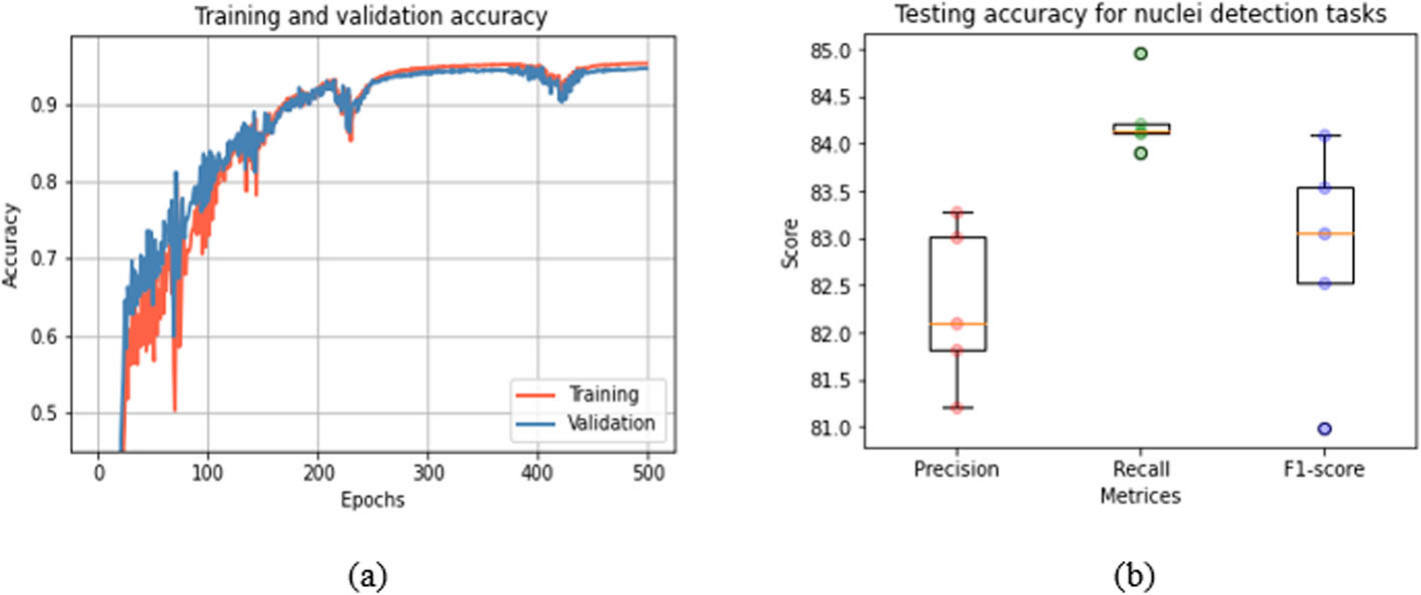
Results: (a) Training and validation accuracy and (b) Testing precision, recall, and F1-score of UD-Net model for nuclei detection tasks

The training and validation accuracy for the UD-Net model is shown in Fig. 14(a). Figure 14(b) demonstrates the box plot of testing precision, recall, and F1-score for the five independent tests. The precision, recall, and F1-score are calculated with automatic counting of ground truth and model prediction of (96 × 96 pixels) input patches. The quantitative results and comparison against existing methods are shown in Table 3. A recently published paper reported a 0.802 F1-score as the highest testing accuracy for nuclei detection [39], whereas the proposed model shows an average F1-score of 0.8284 ± 0.0106 for nuclei detection tasks, which is approximately 2.26% better performance compared to the SC-CNN model [39].

**Table 3:** Nuclei detection accuracy of the proposed model and comparison against existing methods

Methods	Precision	Recall	Mean F1-score
CRImage [16]	0.657	0.461	0.542
CNN [16]	0.783	0.804	0.793
SSAE [46]	0.617	0.644	0.630
LIPSyM [46]	0.725	0.517	0.604
SC-CNN [39] (M = 1)	0.758	0.827	0.791
SC-CNN [39] (M = 2)	0.781	0.823	0.802
Proposed (UD-Net)	**0.822**	**0.842**	**0.828**

**Fig. 15:**
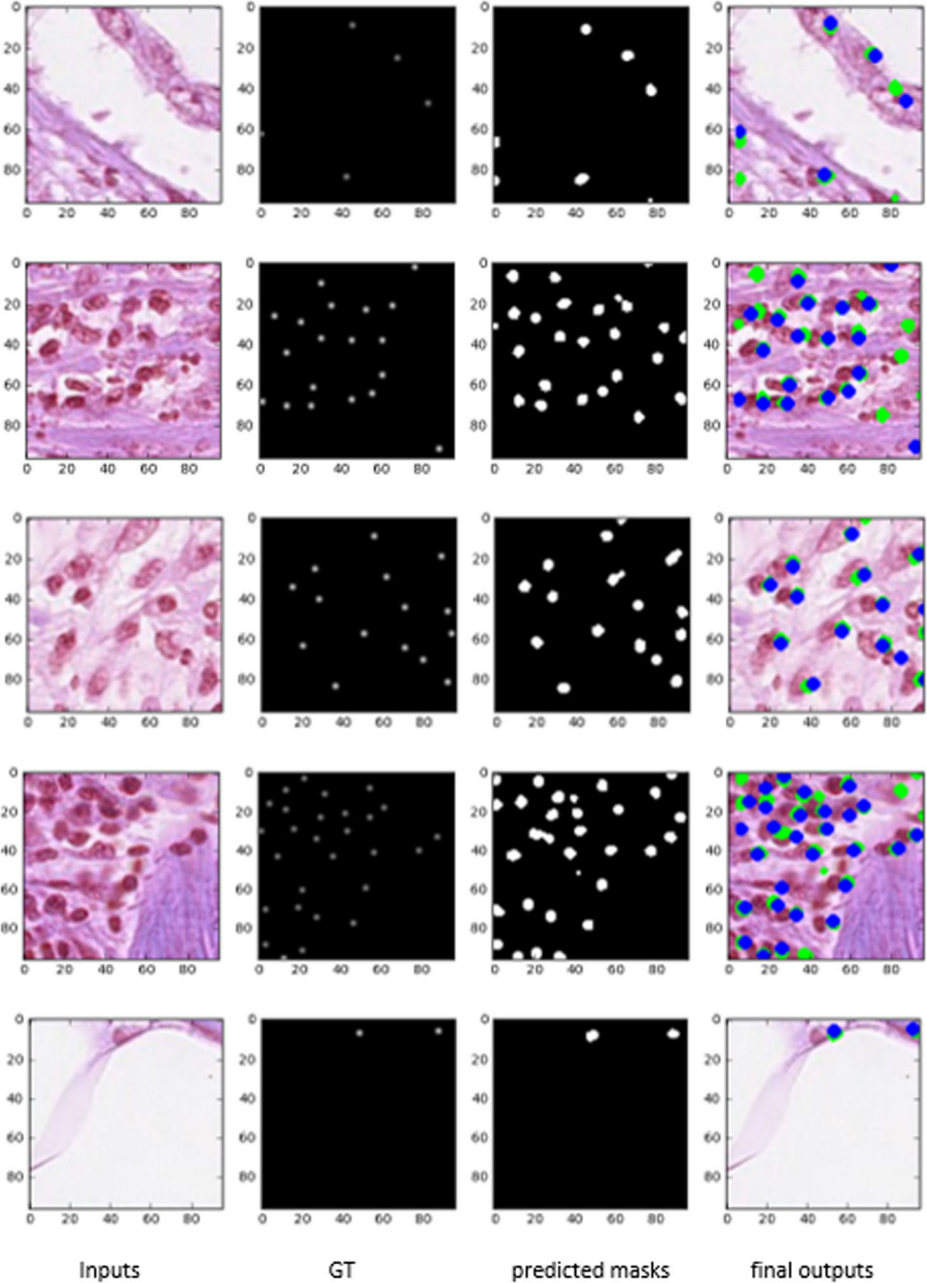
Nuclei detection outputs with inputs, ground truth, model outputs after thresholding, and final outputs with a blue and green dot. The blue dot represents the ground truth and the green dot shows the center pixels of the network outputs

The patch-level (96 × 96 pixels) nuclei detection and ground truth are shown in Fig. 15. The first column shows the input patches, the second column shows the ground truth masks, and the third column represents the model outputs after thresholding with respect to a value of 0.5. Lastly, the fourth column shows the final outputs with blue and green solid circles, where the blue circles indicate the ground truths and the green circles represent the model outputs respectively. Thus, the quantitative results demonstrate that the UD-Net model can detect the nuclei very accurately.

**Fig. 16:**
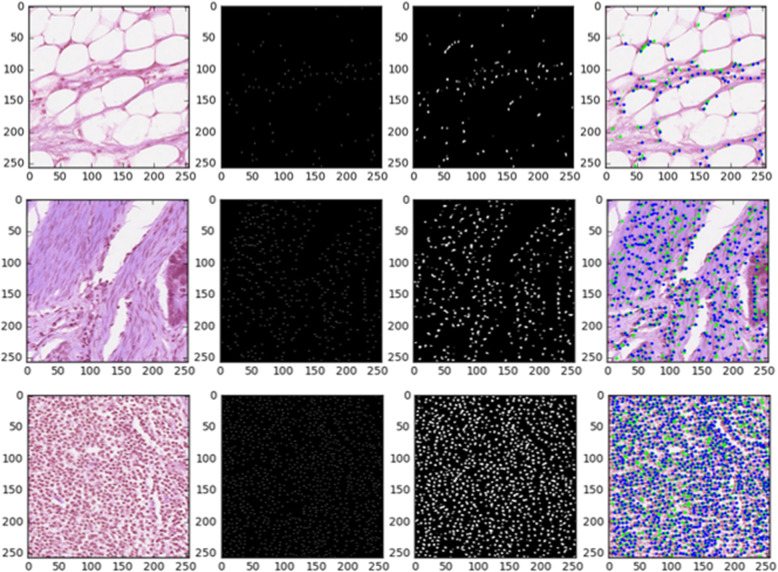
Nuclei detection outputs with large images: the first column shows the input images, the second column shows the ground truth mask, the third column represents the predicted mask by the model, and the fourth column shows the final results where green and blue dots represent the ground truth and detection respectively

After generating the patch-based outputs, we merged all the patches (96 × 96 pixels) to create results for the entire input image (480 × 480 pixels). Figure 16 shows the outputs of 250 × 250 pixels images which are cropped by the merged images of (480 × 480 pixels).

### Analysis

We conducted a set of experiments to evaluate three essential tasks for nuclei classification, segmentation, and detection tasks. First, for classification, we applied DenseNet, and an improved version of DenseNet named the DCRN. The DenseNet provides a performance of 0.7941 in terms of F1-score, whereas the proposed DCRN provides approximately 0.8111 F1-score. The DCRN provides around 1.7% better performance in terms of F1-score against a recently published model of a softmax Convolutional Neural Network (CNN) and a neighboring ensemble predictor (NEP) known as softmax CNN + NEP [39]. Second, we used the R2U-Net for segmentation and achieved 91.90\% testing accuracy, which is around 1.54% better performance than the U-Net model. Third, the UD-Net regression model shows 82.21, 84.27, and 82.8% for precision, recall, and F1-score respectively. The proposed model shows around 2.26% improvement over the existing methods for nuclei detection tasks. Overall, the proposed models provide superior performance for all three tasks. The testing time per sample for classification, segmentation, and detection is shown in Table 4.

**Table 4:** Computational time for the DCRN, R2U-Net, and UD-Net models in the testing phase in seconds

Model	Task	Computational time/epoch (Sec.)
DCRN	Classification	0.0017
R2U-Net	Segmentation	0.57239
UD-Net regression model	Detection	3.19906

## Conclusion

In this study, we proposed three different models, including the Densely Connected Recurrent Convolutional Network (DCRN), the Recurrent Residual U-Net (R2U-Net), and the R2U-Net-based regression named the University of Dayton Net (UD-Net) for nuclei classification, segmentation, and detection tasks respectively. These models are evaluated on three different publicly available datasets. Firstly, we achieved 81.14% testing accuracy in terms of F1-score for the nuclei classification task that is 1.7% higher than recently published results. Secondly, the R2U-Net model shows1.54% better testing accuracy against the U-Net model for nuclei segmentation tasks. Finally, for nuclei detection tasks, we achieved 82.8% testing accuracy in terms of F1-score with the proposed UD-Net, which is a 2.6% better F1-score compared to the existing methods. In the future, we would like to explore these models on more challenging datasets.16

## Data Availability

All of the databases used in this study are publicly available and willing to provide the codes the public on request.

## Abbreviations

DCNN: Densely Connected Neural Network
DCRN: Densely Connected Recurrent Convolutional Network
R2U-Net: Recurrent Residual U-Net
UD-Net: University of Dayton Net
RCC: Routine Colon Cancer
SVM: Support Vector Machine
CAD: Computer Aided Diagnosis
